# Potency of commonly retailed antibiotics in pharmacies found in Adama, Oromia regional state, Ethiopia

**DOI:** 10.1371/journal.pone.0253971

**Published:** 2021-07-01

**Authors:** Demelash Demissie, Teshome Geremew, Adinew Zewdu Chernet, Musa Mohammed Ali

**Affiliations:** 1 Adama Science and Technology University, Adama, Ethiopia; 2 Ethiopian Public Health Institute, Addis Ababa, Ethiopia; 3 College of Medicine and Health Sciences, School of Medical Laboratory Science, Hawassa University, Awasa, Ethiopia; Panjab University, INDIA

## Abstract

**Introduction:**

Antibiotics are commonly used for the treatment and prevention of bacterial infections. The potency of antibiotics can be affected by factors such as temperature, light, moisture, and storage conditions. Inappropriate storage and transportation of antibiotics may lead to loss of potency earlier than the expiry date. The aim of this study was to determine the potency and associated factors of commonly retailed antibiotics.

**Method:**

Institution-based cross-sectional study was conducted on commonly retailed antibiotics in pharmacies that are available in Adama, Ethiopia from March 2018 to June 2018. This study focused on commonly ordered antibiotics such as amoxicillin, azithromycin, ciprofloxacin, and ceftriaxone. Antibiotics to be tested were selected by using a simple random sampling technique. Socio-demographic and related data were collected using a semi-structured questionnaire. Antibiotic susceptibility testing was performed using the disc diffusion method as described in the Clinical Laboratory Standard Institute guideline.

**Results:**

Mean inhibition zones of amoxicillin, ciprofloxacin, azithromycin, and ceftriaxone were 14.2 ± 4 mm, 30.9 ± 4.2 mm, 17.47 ± 3.83 mm, and 32.7±1.8 respectively. Out of 164 antibiotics tested, 61% passed the potency test. The potency of antibiotics varies across different countries in which 53.7% and 54.6 of antibiotics from India and Ethiopia passed the potency test. All ceftriaxone tested in this study passed the potency test. Factors such as air condition of pharmacy (X^2^ = 4.27; *p* = 0.039), source of all antibiotics (X^2^ = 5.41; *p* = 0.02), and source of amoxicillin (X^2^ = 4.73; *p* = 0.03) were significantly associated with potency of antibiotics.

**Conclusions:**

About 40% of antibiotics tested in the current study did not pass the potency test; this warrants further investigation to identify the magnitude of the problem and its causes at a large scale.

## Introduction

Antibiotics have been used for the treatment and prevention of bacterial infections [1]. The effectiveness of antibiotics can be affected by temperature, light, moisture, and storage condition [2]. Counterfeit and substandard antibiotics can also affect the quality of antibiotics. Counterfeit antibiotics are deliberately mislabeled with a fake identity, composition, and source. This can affect generic and branded antibiotics and may include wrong ingredients, no active pharmaceutical ingredients (APIs), and insufficient ingredients. And it also includes antibiotics which are sold with fake labeling and fake packaging [3].

Substandard antibiotics are produced by manufacturers authorized by the national medicine regulatory authority which does not meet the specifications set by national standards [4]. A mild difference in the concentration of the active ingredient during antibiotic preparation may cause allergic reactions, unexpected side effects, poisoning, untreated disease, and early death [5,6]. Careful *in vitro* evaluations are necessary at regular intervals to check the potency of antibiotics sold in the pharmacies [7].

Despite the role of antibiotics in treating the disease; ineffective antibiotics can pose great risks to individuals and even threaten the lives of consumers [8]. Degradation of antibiotics occurs much before they approach the expiry dates because of several factors such as exposure to light and moisture [9]. The loss of potency during storage may influence the efficacy and safety of antibiotics [10].

Poor-quality antibiotics can reach the market through substandard production of legitimate antibiotics due to inadequate quality-control processes during manufacturing, as well as by deliberate fraudulent practices [11]. The wide use of antibiotics with poor quality can lead to the emergence and spread of antibiotic-resistant strains of bacteria in the community [12]. Infection caused by antibiotic-resistant bacteria are more difficult to treat, can lead to long-term illness, increase healthcare costs, and death [9].

Expansion of pharmaceutical industries in many countries with advancement in transportation technologies facilitated not only trade of genuine pharmaceutical products but also the circulation of poor-quality and counterfeited antibiotics in list-income countries [13]. Adama is situated along the road that connects Addis Ababa with Dire-Dawa city. A large number of trucks use the same route to travel to and from the seaports of Djibouti and additionally, the new Addis Ababa-Djibouti railway runs through Adama. This makes Adam one of the busiest cities in the country with strong business activities and several kinds of illegal commodities including counterfeit antibiotics that might be sold in pharmacies found in Adama city. The aim of this study was to determine the potency and associated factors of commonly sold antibiotics in pharmacies that are found in Adama city.

## Materials and methods

### Study setting

This study was conducted in pharmacies located in Adama, Oromia Regional State, Ethiopia. Adama is situated at 99 km to the southeast of Addis Ababa at an altitude of 1712 meters above sea level between the base of an escarpment to the west, and the Great Rift Valley to the East.

### Study design and study population

An institution-based cross-sectional study was conducted on commonly retailed antibiotics from March 2018 to June 2018. In this study, all pharmacies in Adama were included. A total of 164 antibiotics belonging to four categories of antibiotics were included. These antibiotics were amoxicillin (n = 42), ciprofloxacin (n = 42), azithromycin (n = 38), and ceftriaxone (n = 42). From each pharmacy, one antibiotic was randomly selected. Four pharmacies did not have azithromycin. Antibiotics were selected because they are commonly ordered in the study area. Amoxicillin was in the form of a capsule (one stripe contains 10 capsules); ciprofloxacin was in the form of tablets (one stripe contains 10 tablets), and azithromycin was in the form of tablet (one stripe contains 3 tablets). Ceftriaxone was in the form of vials.

### Recommended storage condition of evaluated antibiotics

During the study period, all antibiotics tested in this study were stored in a room with good ventilation and a temperature range of 2–30°C.

### Study variables

Dependent variable: Potency of antibiotics.

Independent variables: Type of pharmacy, air condition of pharmacy, the shelf life of antibiotics, and source of antibiotics.

### Data collection

We developed a semi-structured questionnaire to collect socio-demographic characteristics of dispensers and the condition of antibiotics and pharmacies. From each pharmacy, one pharmacist or druggist was interviewed. All selected antibiotics were checked for batch numbers, manufacturer, and expiry date. After the assessment, antibiotics were transported to the Oromia Public Health Laboratory (OPHL) for antimicrobial susceptibility testing.

### Antibiotic disc preparation

To prepare antibiotic disc we followed the procedure suggested by Clinical Laboratory Standard Institute (CLSI) [14]. Discs with 6 mm diameter were prepared by punching a sheet of Whatman Number 3 filter paper (United Kingdom) using a perforator. To obtain 10 μg of amoxicillin disc, 500 mg of amoxicillin tablet was dissolved in 250 ml of phosphate buffer and 5 μL of dissolved stock antibiotic was impregnated onto the 6 mm sized disc. To obtain 5 μg of ciprofloxacin disc, 500 mg of ciprofloxacin tablet was dissolved in 500 ml of distilled water and 5 μL of dissolved stock antibiotic was impregnated onto the 6 mm sized disc. To obtain 30 μg of ceftriaxone, 1000 mg of injectable ceftriaxone was dissolved in 166.7 ml of distilled water and 5 μL of dissolved stock antibiotic was impregnated onto the 6 mm sized disc. To obtain 15 μg of azithromycin, 500 mg of azithromycin tablet was dissolved in a 166.7 ml of 95% ethanol with a broth media and 5 μL of dissolved stock antibiotic was impregnated onto the 6 mm sized disc (S1 Table in S1 File). To assess the potency of antibiotics, two reference strains such as *Escherichia coli* (ATCC-25922) and *Staphylococcus aureus* (ATCC-25923) were used.

### Antimicrobial susceptibility testing

The antimicrobial susceptibility testing was performed following the Kirby-Bauer disc diffusion method as described in the CLSI [14]. Briefly, the reference strains (*E*. *coli* and *S*. *aureus*) were sub-cultured onto blood agar and incubated at 37°C overnight. Using a sterile wire loop, 3–5 pure colonies were emulsified in 5 mL of normal saline until the turbidity matches 0.5 McFarland standard. Using a sterile dry cotton swab, bacterial suspensions were uniformly inoculated onto the entire surface of Muller Hinton agar (MHA). Antibiotics disks were placed on the surface of MHA and incubated aerobically at 37°C for 16–18 hours. The diameter of the zone of inhibition was measured using a ruler; isolates were classified as potent (pass) or not potent (fail) based on the CLSI cut point [14]. All experiments were conducted in triplicates and the means were recorded to determine the potency of antibiotics.

### Pass criteria based on the susceptibility range set by CLSI

Amoxicillin: It is considered as ‘pass’ if the diameter of the zone of inhibition falls within or greater than the susceptibility range of 15–22 mm.

Ciprofloxacin: It is considered as ‘pass’ if the diameter of the zone of inhibition falls within or greater than the susceptibility range of 30–40 mm.

Azithromycin: It is considered as ‘pass’ if the diameter of the zone of inhibition falls within or greater than the susceptibility range of 21–26 mm.

Ceftriaxone: It is considered as ‘pass’ if the diameter of the zone of inhibition falls within or greater than the susceptibility range of 29–35 mm.

### Data quality control

The semi-questionnaire was validated and pretested before use in the actual study. All laboratory tests were performed according to the CLSI guideline. The sterility and performance of the culture media were checked; the performances of all reagents and materials were checked and ensured. Prior to use, all discs were sterilized and the sterility was checked by placing 8 randomly selected discs on uninoculated MHA agar and incubated at 37°C for 18 hours. Selected discs impregnated with only solvent were checked for antibacterial activity. The disc diffusion method used in the current study was verified by running the method 20 times using standard antibiotics obtained from OPH: Amoxicillin (10 μg), ciprofloxacin (5 μg), azithromycin (15 μg), and ceftriaxone (30 μg). The precisions for amoxicillin, ciprofloxacin, azithromycin, and ceftriaxone were 2.364 mm, 3.364 mm, 1.63 mm, and 1.67 mm respectively. The uncertainty measurement for amoxicillin, ciprofloxacin, azithromycin, and ceftriaxone with a 95% CI were 17–19 mm, 31–32 mm, 21–24 mm, and 31–33 mm respectively.

### Data management and analysis

Data were checked, coded, and entered into the computer using EP INFO version 7 and analyzed by using a Statistical Package for Social Sciences (SPSS) version 20. Data were analyzed using Chi-square (X^2^); a *p*-value of < 0.05 was considered as statistically significant.

## Results

### Socio-demographic data

In this study, a total of 42 pharmacies were included. Out of which, 81% belongs to the private and 19% belongs to the government. A total of 42 dispensers (13 males and 29 females) were interviewed. The ages of 45.2% of dispensers were between 31–40 years. The work experience of 54.8% of dispensers was 5–9 years (Table **1**).

**Table 1:** Characteristics of dispensers and types of Pharmacy at Adama, Oromia, Ethiopia, March 2018 to June 2018 (N = 42).

Variables	Category	n (%)
Type of Pharmacy	Private	34 (81)
	Government	8 (19)
Sex of dispensers	Male	13 (31)
	Female	29 (69)
Age of dispensers in years	≤25	4 (9.5)
	26–30	15 (35.7)
	31–40	19 (45.2)
	41–49	4 (9.5)
	≥50	-
Qualification of dispensers	Druggist	8 (19)
	Pharmacist	34 (81)
Work experience of dispensers in years	<1	-
	1–4	6 (14.3)
	5–9	23 (54.8)
	10–14	9 (21.4)
	≥15	4 (9.5)

#### Characteristics and source of antibiotics

Most amoxicillin was manufactured in Ethiopia (73.8%) whereas most ciprofloxacin was manufactured in South Korea and Romania. Most of the ceftriaxone were manufactured in China (Table **2**).

**Table 2:** Frequency of antibiotics based on manufacturers at Adama, Ethiopia, March 2018 to June 2018.

Antibiotics	Manufacturer	n (%)
Amoxicillin	APF (Ethiopia)	15 (35.7)
	DENK-Pharma (Germany)	1 (2.4)
	EPHARM (Ethiopia)	16 (38.1)
	GlaxoSmithKline (UK)	3 (7.1)
	Kopra Ltd (India)	6 (14.3)
	Remedica (Cyprus)	1 (2.4)
	Total	42 (100)
Ciprofloxacin	APF (Ethiopia)	4 (9.5)
	Brawn Lab (India)	1 (2.4)
	Cadila Pharmaceutical (Ethiopia)	4 (9.5)
	EPHARM(Ethiopia)	6 (14.3)
	Huonsco Ltd (S. Korea)	10 (23.8)
	Leben laboratories (India)	7 (16.7)
	Sandoz a Navartis Company (Romania)	10 (23.8)
	Total	42 (100)
Azithromycin	Bafna Pharmaceutical Ltd (India)	1 (2.6)
	Beximco pharmaceutical Ltd (Bangladesh)	7 (18.4)
	Cadila Pharmaceuticals (Ethiopia)	9 (23.7)
	Coral Laboratories Ltd (India)	2 (5.3)
	Corporation Ltd (Kenya)	1 (2.6)
	Deva Holding (Turkey)	5 (13.2)
	EPHARM (Ethiopia)	1 (2.6)
	Sandoz a Navartis company (Romania)	2 (5.3)
	Sherya life science (India)	1 (2.6)
	Umedica Laboratories Ltd (India)	1 (2.6)
	ZIM Laboratories Ltd (India)	8 (21.1)
	Total	38 (100)
Ceftriaxone	Ashish Life Science (India)	6 (14.3)
	AsralSteriTech PLC Ltd (India)	5 (11.9)
	BilimPharmaceutical (Turkey)	2 (4.8)
	CSPC ZhougnuoPharm (China)	9 (21.4)
	Gulf Pharmaceuticalind (UAE)	3 (7.1)
	Shandonglukang pharmaceutical (China)	13 (30.9)
	Theon Pharmaceutical (India)	2 (4.8)
	VHB medi science ltd (India)	1 (2.4)
	Zhuhai Kinhoo pharmaceutical (China)	1 (2.4)
Total		42 (100)

EPHARM: Ethiopian pharmaceutical manufacturing, APF: Addis pharmaceutical factory plc (private limited company), UAE: United Arab Emirates, UK: United Kingdom.

### Shelf life and handling conditions of antibiotics

Almost all antibiotics retailed in the private and government pharmacies in Adama had a long expiry date (> six months). At the time of collection, none of the antibiotics were expired. All amoxicillin and ciprofloxacin had a long expiry date. Thirty (78.9%) and 38 (90.5) azithromycin and ceftriaxone had a long expiry date respectively. Forty (90.5%) of the pharmacies had enough storage area. Out of 42 pharmacies, 25 (59.5%) had ventilator with good working conditions, 32 (76.2%) had refrigerator and 26 (61.9%), and 25 (59.5%) of them had a thermometer in dispenser and store room respectively. Thirty-eight (90.5%) of the respondents replied that there was an inspection by a regulatory body. Thirty-one (81.6%) of respondents replied that inspection was made in three months intervals and 7 (18.4%) replied that inspection was made in six months intervals.

### Mean inhibition zones of antibiotics

Mean inhibition zones of amoxicillin, ciprofloxacin, azithromycin, and ceftriaxone were 14.2 ± 4 mm, 30.9 ± 4.2 mm, 17.47 ± 3.83 mm, and 32.7±1.8 respectively. Most of the amoxicillin from Ethiopia (EPHARM-Ethiopia) had a mean zone of inhibition of less than 12 mm (Table 3).

**Table 3:** Mean zone of inhibition of selected antibiotics collected from pharmacies found in Adama, Ethiopia, March 2018 to June 2018.

Bacteria	Amoxicillin manufacture	n	Mean ZI (mm±SD)	Standard range(mm)
*E*. *coli* **(***E*. *coli* ATCC 25922)	APF-Ethiopia	15	14.73±3.4	15–22
	DENK Pharma-Germany	1	15 ±0	
	EPHARM-Ethiopia	16	11.88±4.7	
	GSK-London	3	14.67± 3.2	
	KOPRA LIMTED-India	6	21.33±2.3	
	Remedica-Cyprus	1	15±0	
	Mean IZ of Amoxicillin	42	14.2±4.0	
	**Ciprofloxacin manufacturer and country**			
*E*. *coli* (ATCC 25922)	APF-Ethiopia	4	34±7.1	30–40
	BRAWN Lab.-India	1	32±0	
	CADILA Pharmaceutical- Ethiopia	4	34±2.8	
	EPHARM-Ethiopia	6	32.5±4.6	
	Huonsco Ltd- S. korea	10	31.1±1.9	
	Leben lab.-India	7	26.3±8.2	
	SANDOZ aNavartis-Romania	10	29.1±3.5	
	Mean IZ of ciprofloxacin	42	30.9±4.2	
	**Azithromycin manufacture and country**			
*S*. *aureus* (ATCC 25923)	Bafna Pharmaceutical-India	1	12±0	21–26
	Beximico pharmaceutical -Bangladesh	7	16.5±4.57	
	CADILA Pharmaceutical -Ethiopia	9	18.5±3.5	
	Coral Lab.-India	2	19.33±4.18	
	Corporation limited-Kenya	1	20±0	
	Deva Holding-Turkey	5	19.33±4.18	
	EPHARM-Ethiopia	1	14±0	
	Sandoz–Romania	2	17±4.2	
	SHERYA LIFE Science- India	1	20±0	
	Umedica Laboratory-India	1	14±0	
	ZIM lab.-India	8	16.12±3.5	
	Mean ZI of Azithromycin	38	17.47±3.83	
	**Ceftriaxone manufacturer and country**			
*E*. *coli* (ATCC 25922)	Ashish life science-India	6	33±1.7	29–35
	Asralsteril Tech-India	5	32.4±1.3	
	BILIM pharmaceutical- Turkey	2	34±1.4	
	CSPC Zhougnuo Pharmaceutical-China	9	33.2±1.4	
	Gulf pharmaceutical industry-UAE	3	32±1.7	
	Shandonglukang pharmaceutical-China	13	32.5±3	
	Theon Pharmaceutical-India	2	32±1.4	
	VHB MEDI Science limited-India	1	35±0	
	Zhuhai Kinhoo pharmaceutical- China	1	33±0	
	Mean IZ of ceftriaxone	42	32.7±1.8	

Mm: millimeter, SD: Standard deviation, ZI: Zone of inhibition, ATCC: American type culture collection, UAE: United Arab Emirates.

### Potency of antibiotics

Out of 164 antibiotics tested in this study, 61% were potent. 53.7% and 54.6 of antibiotics from India and Ethiopia were potent respectively (Table **4**). The total potency of amoxicillin, ciprofloxacin, azithromycin, and ceftriaxone were 50%, 66.7%, 23.7%, and 100% respectively (Table 5).

**Table 4:** Potency of selected antibiotics collected from pharmacies found in Adama, Ethiopia based on the source country, March 2018 to June 2018.

Country	Pass n (%)	Fail, n (%)	Total
India	22 (53.7)	19 (46.3)	41
Bangladesh	-	7 (100)	7
Ethiopia	30 (54.6)	25 (45.5)	55
Kenya	-	1 (100)	1
Turkey	6 (85.7)	1(14.3)	7
Romania	3 (25)	9 (75)	12
Germany	1 (100)	-	1
United Kingdom	2 (66.7)	1 (33.3)	3
Cyprus	1 (100)	-	1
S. Korea	9 (90)	1 (10)	10
China	23 (100)	-	23
UAE	3 (100)	-	3
**Total**	**100 (61)**	**64 (39)**	**164**

UAE: United Arab Emirates, S. Korea: South Korea.

**Table 5:** Potency of selected antibiotics collected from pharmacies found in Adama, Ethiopia based on the manufacturer and country, March 2018 to June 2018.

**Amoxicillin**						
Manufacturer	Country	Brand name	Concentration	Pass, n (%)	Fail, n (%)	Total
APF	Ethiopia	Amoxid	500mg	6(40)	9 (60)	15
DENK Pharma	Germany	Amoxi-Denk	500mg	1 (100)	-	1
EPHARM	Ethiopia	Amoxicillin	500mg	8 (50)	8 (50)	16
GlaxoSmithKline	UK	Amoxil	500mg	2 (66.7)	1 (33.3)	3
KOPRA limited	India	AMYN	500mg	3 (50)	3(50)	6
Remedica	Cyprus	Amoxapen	500mg	1 (100)	-	1
Total				21 (50)	21 (50)	42
**Ciprofloxacin**						
APF	Ethiopia	CIF LOX	500mg	3 (75)	1 (25)	4
BRAWN Laboratory	India	Brucipro	500mg	1 (100)	-	1
CADILA Pharmaceutical	Ethiopia	Ciprodac	500mg	4 (100)	-	4
EPHARM	Ethiopia	Ciprofloxacin	500mg	5 (88.3)	1 (16.7)	6
Huonsco Ltd	S. Korea	Floxine	500mg	9 (90)	1 (10)	10
Leben laboratory	India	Ciproleb	500mg	3 (42.9)	4 (57.1)	7
SANDOZ aNavartis comp	Romania	Serviflox	500mg	3 (30)	7 (70)	10
Total				28 (66.7)	14 (33,3)	42
**Azithromycin**						
Bafna Pharmaceutical	India	AZIBIAL	500mg	-	1 (100)	1
Beximico pharmaceutical	Bangladesh	Azithrocin	500mg	-	7(100%)	7
CADILA Pharmaceutical	Ethiopia	Zycin	500mg	4 (44.4)	5 (55.6)	9
Coral Laboratory	India	Cortzite	500mg	-	2 (100)	2
Corporation limited	Kenya	THROZA	500mg	-	1 (100)	1
Deva Holding	Turkey	Azitro	500mg	4 ((80)	1 (20)	5
EPHARM	Ethiopia	Ephazit	500mg	-	1 (100)	1
Sandoz	Romania	Binozyt	500mg	-	2 (100)	2
SHERYA LIFE Science	India	ZIT	500mg	-	1 (100)	1
Umedica Laboratory	India	UZET	500mg	-	1 (100)	1
ZIM Laboratory	India	Azito	500mg	1 (12.5)	7 (87.5)	8
Total				9 (23.7)	29 (76.3)	38
**Ceftriaxone**						
Ashish life science	India	CEFTASH	1000mg	6 (100)	-	6
Asralsteril Tech	India	ZEFONE100	1000mg	5 (100	-	5
BILIM pharmaceutical	Turkey	FORSEF	1000mg	2 (100)	-	2
CSPC Zhougnuo Pharmaceutical	China	Ceftazone	1000mg	9 (100)	-	9
Gulf pharmaceutical industry	UAE	Triaxon	1000mg	3 (100)	-	3
Shandonglukang pharmaceutical	China	Ceftriaxone	1000mg	13 (100)	-	13
Theon Pharmaceutical	India	THEOXONE	1000mg	2 (100)	-	2
VHB MEDI Science limited	India	IVIXONE	1000mg	1 (100)	-	1
Zhuhai Kinhoo pharmaceutical	China	Ceftriaxone	1000mg	1 (100)	-	1
Total				42 (100)	-	42

UAE: United Arab Emirates, UK: United Kingdom, APF: Addis pharmaceutical factory.

### Factors that influence the potency of antibiotics

In the current study, among different factors assessed air condition of the pharmacy for amoxicillin (X^2^ = 4.27; *p* = 0.039), the source of all antibiotics (X^2^ = 5.41; *p* = 0.02), and the source of amoxicillin (X^2^ = 4.73; *p* = 0.03) were significantly associated with potency of antibiotics (Table **6**).

**Table 6:** Factors that affect the potency of selected antibiotics collected from pharmacies found in Adama, Ethiopia (March 2018 to June 2018).

Variables		Potency of antibiotics*		X^2^ value	*p*-value
		Pass, n (%)	Fail, n (%)		
**Type of pharmacy from where all antibiotics obtained**	**Private**	**49 (48)**	**53 (52)**	**3.47**	**0.07**
	**Government**	**14 (70)**	**6 (30)**		
Type of pharmacy from where amoxicillin was obtained	Private	18 (52.9)	16 (47.1)	0.24	0.63
	Government	5 (62.5)	3 (37.5)		
Type of pharmacy from where ciprofloxacin was obtained	Private	20 (58.8)	14 (41.2)	3.68	0.06
	Government	8 (100)	-		
Type of pharmacy from where azithromycin was obtained	Private	11 (32.4)	23 (67.6)	0.09	0.77
	Government	1 (25)	3 (75)		
**Air condition of pharmacy for all antibiotics**	**Ventilation**	**40 (55.6)**	**32 (44.4)**	**1.07**	**0.30**
	**No ventilation**	**23 (46)**	**27 (54)**		
Air condition of pharmacy for amoxicillin	Ventilation	17 (68)	8 (32)	4.27	0.039
	No ventilation	6 (35.3)	11 (64.7)		
Air condition of pharmacy for ciprofloxacin	Ventilation	16 (64)	9 (36)	0.19	0.66
	No ventilation	12 (70.6)	5 (29.4)		
Air condition of pharmacy for azithromycin	Ventilation	7 (31.8)	15 (68.2)	0.001	0.97
	No ventilation	5 (31.3)	11 (68.7)		
**Shelf life for all antibiotics**	Long expired date	84 (55.3)	68 (44.7)	0.16	0.69
	Near to expiry (<6mnth)	4 (33.3)	8 (66.7)		
Shelf life for azithromycin	Long Expiry date	9 (30)	21 (70)	0.160	0.69
	Near to expiry date (<6mnth)	3 (37.5)	5 (62.5)		
**Source for all antibiotics**	Local	29 (53.7)	25 (46.3)	5.41	**0.02**
	Foreign	76 69.1)	34 (30.9)		
Source for amoxicillin	Local	14 (45.2)	17 (54.8)	4.73	0.03
	Foreign	9 (81.8)	2 (18.2)		
Source for ciprofloxacin	Local	11 (84.6)	2 (15.4)	2.97	0.85
	Foreign	17 (58.6)	12 (41.4)		
Source for azithromycin	Local	4 (40)	6 (60)	1.67	0.51
	Foreign	8 (28.6)	20 (71.4)		
Awareness on unlicensed antibiotic distributer	Yes	42 (53.2)	37 (46.8)	0.001	0.97
	No	46 (54.1)	39 (45.9%)		
Work experience	< 10years	64 (52.9)	57 (47.1)	0.023	0.88
	≥10 years	24(55.8)	19 (44.2%)		

* Ceftriaxone was excluded from analysis because all of them (n = 42) were potent.

## Discussion

Out of 164 antibiotics tested in this study, only 61% were found to be potent. The potency of antibiotics varies based on the countries of origin in which 53.7% and 54.6% of antibiotics from India and Ethiopia were potent respectively. Among the four categories of antibiotics tested, less than 50% of azithromycin were not potent with a mean inhibition zone diameter of 17.47±3.83 mm. The inhibition zone was unsatisfactory according to the standard inhibition zone set by the CLSI guideline (21–26 mm) [15]. The inhibition zone of azithromycin in this study was probably affected by the pH of the microbiological growth medium and its dissolubility in 95% ethanol alcohol diluents [16].

About 50% of amoxicillin and ciprofloxacin in the current study were not potent indicating some defect either in the antibiotics itself, storage, limitation of our laboratory methods, and antibiotics can be counterfeit or of substandard [17]. Similarly, bacteria isolated from the clinical specimen was reported to have reduced susceptibility to amoxicillin and ciprofloxacin [18,19]. Unlike this study, most of the amoxicillin tested in the Middle East was not counterfeit and contained the amount of ingredients claimed by the manufacturer [20,21].

This study revealed that the mean inhibition zone diameter of amoxicillin to be 14.2 ± 4 mm which is not in line with the finding reported from Ghana (16 ± 0.6mm) [22]. Moreover, most of the amoxicillin produced in Ethiopia (EPHARM) had a mean zone of inhibition of less than 12 mm which is below the standard set by the CLSI. The variation observed could be due to a difference in the manufacturer; most of the amoxicillin in this study was locally produced. The difference could also be attributed to the laboratory method used to determine the potency of antibiotics.

The mean inhibition zone of ciprofloxacin was 30.9 ± 4.2mm which is in the range set by CLSI. However, most of them were not potent. In this study, the mean inhibition zone of ceftriaxone was 32.7±1. According to CLSI inhibition zone range, all ceftriaxone antibiotics were potent. Several studies, which were conducted on different clinical isolates, indicated the effectiveness of ceftriaxone [18,19,23].

The potency of antibiotics can be affected by various factors. In the present study, the air condition of the pharmacy (X^2^ = 4.27; *p* = 0.039) and source of the antibiotics were significantly associated with the potency of antibiotics (X^2^ = 5.41; *p* = 0.02). According to this study, 52% of all antibiotics from private pharmacies were not potent. About 64% of amoxicillin stored in a pharmacy with no ventilator failed to pass the potency test indicating the importance of the ventilator in keeping the integrity of antibiotics stored in the pharmacy. Most of the antibiotics (46.3%) from the local source (Ethiopia) were not potent; specifically, 54.8% of amoxicillin from the local source was unable to pass the potency test (X^2^ = 4.73; *p* = 0.03). This could be due to problems that could arise during the production, shipment, and storage condition of antibiotics. Moreover, antibiotics could be of substandard or counterfeit which may contain active ingredients below the required level.

### Limitation of the study

Only four antibiotics were tested, which might not be representative of other antibiotics. We faced a shortage of similar studies to compare the findings of this study.

## Conclusions

About 60% of antibiotics tested in the current passed the potency test. The potency of antibiotics varies based on the source country where 53.7% and 54.6 of antibiotics from India and Ethiopia passed the potency test. Most of the azithromycin did not pass the potency test while all ceftriaxone passed the potency test. Among factors assessed, air condition of pharmacy, source of all antibiotics, and source of amoxicillin were significantly associated with potency of antibiotics. This study highlights the importance of further investigation to identify the magnitude of the problem and its causes at a large scale.

## Supporting information

S1 File (DOCX)

## Abbreviations

CLSI: Clinical Laboratory Standard Institute
OPHL: Oromia Public Health Laboratory
ATCC: American type culture collection
SPSS: Statistical Package for Social Sciences
