# Supplementary material for: Potency of commonly retailed antibiotics in pharmacies found in Adama, Oromia regional state, Ethiopia
Source: PLoS One. 2021 Jul 1;16(7):e0253971. doi: 10.1371/journal.pone.0253971 (PMC8248621; doi:10.1371/journal.pone.0253971)
Supplement: S1 File — (DOCX) [file pone.0253971.s001.docx]

**Supplement file**

**Supplement table 1.** Antibiotics dilution, concentration and dispensing volume preparation

| Antibiotics | M | V | C=M/V | P | m=p*c |
| --- | --- | --- | --- | --- | --- |
| Amoxicillin | 500mg | 250ml phosphate buffer PH-6 | 2mg/ml=2μg/ μL | 5 μL | $10\mu g$ |
| Ciprofloxacin | 500mg | 500ml | 1mg/ml=1μg/ μL | 5 μL | $5\mu g$ |
| Azithromycin | 500mg | 161.7ml+5ml of 95% ethanol=166.7ml | 2.9mg/ml=2.9μg/ μL | 5 μL | $15\mu g$ |
| Ceftriaxone | 1000mg | 166.7ml | 5.9mg/ml=5.9μg/ μL | 5 μL | $30\mu g$ |

$$V=\frac{P*M}{m}$$

$$C=\frac{M}{V}$$

Where

- V=Diluent volume to prepare standard concentration
- P=dispensing volume on the disc
- M=gram of antibiotic purchased from pharmacies
- m=standard disc gram based on CLSI 2016
- C= prepared concentration of antibiotics

**Supplement Table 2.** Inhibition zone of selected antibiotics and its status against the standard inhibition zone.

| **Code** | **Azithromycin** | **Antibiotic Efficacious status** | **Amoxicillin** | **Antibiotic Efficacious status** | **Ciprofloxacin** | **Antibiotic Efficacious status** | **Ceftriaxone** | **Antibiotic Efficacious status** |
| --- | --- | --- | --- | --- | --- | --- | --- | --- |
|  | **Acceptable range (21-26mm)** |  | Acceptable range (15-22mm) |  | Acceptable range (30-40mm) |  | Acceptable range (29-35mm) |  |
|  | **Mean Measured value(mm)**  **+SD** |  | **Mean Measured value(mm)**  **+SD** |  | **Mean Measured value(mm)**  **+SD** |  | **Mean Measured value(mm)**  **+SD** |  |
| 1 | 21 | Pass | 14 | Fail | 34 | Pass | 30 | Pass |
| 2 | 13 | Fail | 16 | Pass | 31 | Pass | 34 | Pass |
| 3 | 16 | Fail | 15 | pass | 34 | Pass | 33 | Pass |
| 4 | 14 | Fail | 21 | Pass | 27 | Fail | 34 | Pass |
| 5 | 13 | Fail | 10 | Fail | 21 | Fail | 30 | Pass |
| 6 | 14 | Fail | 11 | Fail | 11 | Fail | 30 | Pass |
| 7 | 14 | Fail | 11 | Fail | 33 | Pass | 30 | Pass |
| 8 | 13 | Fail | 11 | Fail | 30 | Pass | 34 | Pass |
| 9 | 12 | Fail | 17 | Pass | 33 | Pass | 33 | Pass |
| 10 | 13 | Fail | 10 | Fail | 29 | Fail | 30 | Pass |
| 11 | 19 | Fail | 10 | Fail | 32 | Pass | 31 | Pass |
| 12 | 21 | Pass | 21 | Pass | 30 | Pass | 33 | Pass |
| 13 | 18 | Fail | 20 | Pass | 33 | Pass | 33 | Pass |
| 14 | 22 | Pass | 20 | Pass | 33 | Pass | 34 | Pass |
| 15 | 14 | Fail | 16 | Pass | 32 | Pass | 33 | Pass |
| 16 | 14 | Fail | 11 | Fail | 32 | Pass | 33 | Pass |
| 17 | 14 | Fail | 16 | Pass | 33 | Pass | 33 | Pass |
| 18 | 15 | Fail | 15 | pass | 32 | Pass | 33 | Pass |
| 19 | 14 | Fail | 14 | Fail | 32 | Pass | 33 | Pass |
| 20 | 14 | Fail | 11 | Fail | 26 | Fail | 33 | Pass |
| 22 | 16 | Fail | 9 | Fail | 33 | Pass | 30 | Pass |
| 24 | 22 | Pass | 14 | Fail | 28 | Fail | 34 | Pass |
| 28 | 14 | Fail | 14 | Fail | 27 | Fail | 33 | Pass |
| 29 | 17 | Fail | 16 | Pass | 28 | Fail | 33 | Pass |
| 30 | 14 | Fail | 24 | Pass | 26 | Fail | 33 | Pass |
| 31 | 21 | Pass | 6 | Fail | 26 | Fail | 33 | Pass |
| 32 | 17 | Fail | 16 | Pass | 26 | Fail | 30 | Pass |
| 34 | 21 | Pass | 18 | Pass | 26 | Fail | 30 | Pass |
| 35 | 13 | Fail | 14 | Fail | 25 | Fail | 30 | Pass |
| 36 | 17 | Fail | 20 | Pass | 30 | Pass | 33 | Pass |
| 37 | 16 | Fail | 16 | Pass | 27 | Fail | 33 | Pass |
| 38 | 18 | Fail | 9 | Fail | 30 | Pass | 35 | Pass |
| 39 | 17 | Fail | 17 | Pass | 32 | Pass | 35 | Pass |
| 40 | 21 | Pass | 9 | Fail | 32 | Pass | 35 | Pass |
| 42 | 14 | Fail | 15 | pass | 34 | Pass | 30 | Pass |
| 43 | - | - | 7 | Fail | 34 | Pass | 33 | Pass |
| 44 | 23 | Pass | 14 | Fail | 40 | Pass | 34 | Pass |
| 45 | 12 | Fail | 6 | Fail | 40 | Pass | 35 | Pass |
| 46 | - | - | 15 | pass | 38 | Pass | 34 | Pass |
| 47 | - | - | 20 | Pass | 30 | Pass | 33 | Pass |
| 48 | - | - | 6 | Fail | 40 | Pass | 40 | Pass |
| 49 | 21 | Pass | 17 | Pass | 34 | Pass | 35 | Pass |

**Semi-structured Questionnaire**

The following interview and observational questions were designed to collect data from pharmacy dispensers found in both Private and Governmental pharmacies that will help to screen the effectiveness of commonly retailed antibiotics found in Adama City.

1. In what kind of pharmacy are you working currently?

Private Governmental

1. How many employees are working in the pharmacy? [][]

2.1 Number of druggists [][]

2.2. Number of Pharmacist [][]

2.3. Number of other employees [][]

3. Sex of participant

Male Female

1. Age of participants in years [][]
2. Educational level of participants

Level IV Diploma Bachelor’s degree Master’s degree Doctorate

1. Work experience of the participant

Less than 1 year 1-4 years 5-9 years 10-14 years Above 15 years

1. Observe presence of daily temperature record format for
   1. Dispenser rooms: Yes No
   2. Store room: Yes No
   3. Refrigerator: Yes No
2. Is air conditioning of pharmacy in a good working condition?

Yes No

1. Is the store area large enough to allow for order arrangement and proper stock rotation?

Yes No

1. Is there any regulation in antibiotics by regulatory body?

Yes No

1. If your answer is yes for question #9

how often do they regulate? ………………………

1. Are you aware of unlicensed antibiotic distributer?

Yes No

1. Country where antibiotics are manufactured___________________
2. Manufacturer’s name _____________________
3. Concentration of each antibiotics___________
4. Does the pharmacy have functional ventilator?

Yes No

1. Expiry date of each antibiotics_____________
2. Does the pharmacy have refrigerator?

Yes No

1. Does the pharmacy have thermometer in dispenser room?

Yes No

1. Does the pharmacy have thermometer in store room?

Yes No
