# Supplementary material for: Potency of commonly retailed antibiotics in pharmacies found in Adama, Oromia regional state, Ethiopia
Source: PLoS One. 2021 Jul 1;16(7):e0253971. doi: 10.1371/journal.pone.0253971 (PMC8248621; doi:10.1371/journal.pone.0253971)
Supplement: Attachment — Submitted filename: Response to reviewers comments.docx [file pone.0253971.s002.docx]

[Note: HTML markup is below. Please do not edit.]

Reviewers' comments:

Reviewer's Responses to Questions

**Comments to the Author**

1. Is the manuscript technically sound, and do the data support the conclusions?

The manuscript must describe a technically sound piece of scientific research with data that supports the conclusions. Experiments must have been conducted rigorously, with appropriate controls, replication, and sample sizes. The conclusions must be drawn appropriately based on the data presented.

Reviewer #1: No

Reviewer #2: Partly

2. Has the statistical analysis been performed appropriately and rigorously?

Reviewer #1: No

Reviewer #2: I Don't Know

3. Have the authors made all data underlying the findings in their manuscript fully available?

The [PLOS Data policy](http://www.plosone.org/static/policies.action#sharing) requires authors to make all data underlying the findings described in their manuscript fully available without restriction, with rare exception (please refer to the Data Availability Statement in the manuscript PDF file). The data should be provided as part of the manuscript or its supporting information, or deposited to a public repository. For example, in addition to summary statistics, the data points behind means, medians and variance measures should be available. If there are restrictions on publicly sharing data—e.g. participant privacy or use of data from a third party—those must be specified.

Reviewer #1: Yes

Reviewer #2: Yes

4. Is the manuscript presented in an intelligible fashion and written in standard English?

PLOS ONE does not copyedit accepted manuscripts, so the language in submitted articles must be clear, correct, and unambiguous. Any typographical or grammatical errors should be corrected at revision, so please note any specific errors here.

Reviewer #1: No

Reviewer #2: No

5. Review Comments to the Author

Please use the space provided to explain your answers to the questions above. You may also include additional comments for the author, including concerns about dual publication, research ethics, or publication ethics. (Please upload your review as an attachment if it exceeds 20,000 characters)

Reviewer #1: In this submitted manuscript   authors  claimed that about 40% of antibiotics tested in the present study were not potent and the potency of antibiotics varies based on the source of the country.  Authors also claimed that most of azithromycin were not potent while all ceftriaxone were potent.  Author (s) mentioned  that the physicochemical condition in the  pharmacy setup significantly associated with the potency of antibiotics. Manuscript is not technically sound due to poor presentation and   so it is very difficult  to understand what kind of message to give the author (s) in the present MS.

**Response:** Thank you for your valuable comments.

- In order to address the technical issue of the manuscript, we have included the following information in the method section: categories of antibiotics tested and their form (in the study design and population), Recommended storage condition of evaluated antibiotics, antibiotic disc preparation heading with detail description, antimicrobial susceptibility testing heading with detail description, pass criteria heading with detail description. We have also included details of what we did to maintain the quality of the data under Data quality control. All these are shown in the manuscript with track change.

However, there are following  points that need to be addressed :1. What could be the scientific reason,  reduction in potency of antibiotic.?2. Does variation of API ( Source of API) in the antibiotics preparations responsible for antibiotic potency.3. Does the pharmacy set up awareness of this  type of loss in the potency of antibiotics?

**Response:**

- Yes, most of the antibiotics tested did not pass the potency test; several factors can be the reason for the failure. In addition to factors that are significantly associated with the potency of the antibiotics in the current study, other factors such as substandard, counterfeit, laboratory methods used can also affect the potency of antibiotics. There are also possibilities of illegal antibiotics in pharmacies. We have mentioned these reasons in the middle and as the end of the discussion section.
- The pharmacies are of about the ineffectiveness of antibiotics indirectly from customers (there is no improvement despite antibiotic intake). We have communicated the finding of this study to concerned bodies.

Reviewer #2: Comments
This study about “Potency of commonly retailed antibiotics in pharmacies found in Adama, Oromia regional state, Ethiopia -” provides interesting data but has many shortcomings. These points should be addressed. My major comments are as follows.
1. The manuscript is not written so well and therefore, it is difficult to understand what authors want to say.

**Response:** Thank you for your constructive comments.

- We agree with comment; we have revised the manuscript as shown in the track change.

2.Language of the manuscript needs to be improved thoroughly. Some points, words or sentences have been highlighted in the manuscript and corrections should be made throughout the manuscript

**Response:**

- We agree with comment; we have revised the languages as shown in the track change.

3.On page number 13, E. coli must be italicized in table 3.

**Response:** We italicized as suggested.

4. “To obtain 10 μg of amoxicillin disc, 500 mg of amoxicillin tablet was dissolved in 250 ml of phosphate buffer and 5 μm of dissolved stock antibiotic was impregnated on the 6 mm sized disc. To obtain 5 μg of ciprofloxacin disc, 500mg of ciprofloxacin tablet was dissolved in 500ml of distilled water and 5 μm of dissolved stock antibiotic was impregnated on the 6 mm sized disc. To obtain 30 μg of ceftriaxone, 1000mg of injectable ceftriaxone was dissolved in 166.7ml of distilled water and 5 μm of dissolved stock antibiotic was impregnated on the 6 mm sized disc. To obtain 15 μg of azithromycin, 500mg of azithromycin the tablet was dissolved in a 166.7 ml of 95% ethanol with a broth media and 5 μm of dissolved stock antibiotic was impregnated on the 6 mm sized disc.” Authors have used different volumes of different solvents and different amounts of antibiotics, justify.

**Response:** For preparation of antibiotics, we have followed CLSI guideline (CLSI guideline M_100S26, 2016, 26 ed, Table 6A, page 192). Preparation procedure is attached as supplement file.

5.“5 μm of dissolved stock antibiotic was impregnated on the 6 mm sized disc”. What does “5 μm” mean? How authors confirmed/checked that 5 μm was impregnated on disc?

**Response:** We agree with the comment and corrected as 5 μL.

6.Present work describe the potency of antibiotics. In Table 4, what factors were considered to define/calculate potency of antibiotics on the basis of country?

**Response:** We agree with the comment and included ‘pass’ and ‘fail’ criteria set by CLSI in the method section as shown in the track change.

7.Which factors define the pass and fail percentage of any antibiotic in Table 4 and 5?

**Response:** We agree with the comment and included ‘pass’ and ‘fail’ criteria set by CLSI in the method section.

6. PLOS authors have the option to publish the peer review history of their article ([what does this mean?](https://journals.plos.org/plosone/s/editorial-and-peer-review-process#loc-peer-review-history)). If published, this will include your full peer review and any attached files.


If you choose “no”, your identity will remain anonymous but your review may still be made public.


**Do you want your identity to be public for this peer review?** For information about this choice, including consent withdrawal, please see our [Privacy Policy](https://www.plos.org/privacy-policy).

Reviewer #1: **Yes:** NA

Reviewer #2: No

[NOTE: If reviewer comments were submitted as an attachment file, they will be attached to this email and accessible via the submission site. Please log into your account, locate the manuscript record, and check for the action link "View Attachments". If this link does not appear, there are no attachment files.]

While revising your submission, please upload your figure files to the Preflight Analysis and Conversion Engine (PACE) digital diagnostic tool, <https://pacev2.apexcovantage.com/>. PACE helps ensure that figures meet PLOS requirements. To use PACE, you must first register as a user. Registration is free. Then, login and navigate to the UPLOAD tab, where you will find detailed instructions on how to use the tool. If you encounter any issues or have any questions when using PACE, please email PLOS at [figures@plos.org](mailto:figures@plos.org). Please note that Supporting Information files do not need this step.
